# Medical students’ perception of paid E-learning

**DOI:** 10.1371/journal.pone.0317340

**Published:** 2025-03-10

**Authors:** Parsa Mohri, Yilmaz Onat Koyluoglu, Mustafa Ege Seker, S‚ehla Nurefs‚an Sancak, Mirkan Dikeç, Ayça Aydemir Mazlumoglu, Yesim Gurol, Ilhan Cem Sungur

**Affiliations:** 1 Acıbadem Mehmet Ali Aydinlar University, School of Medicine, Istanbul, Turkey; 2 Acıbadem Mehmet Ali Aydinlar University Library, Istanbul, Turkey; 3 Medical Education Department, Acıbadem Mehmet Ali Aydinlar University, Istanbul, Turkey; College of Medical Sciences, NEPAL

## Abstract

**Background:**

In addition to the medical school curriculum, medical students often take the initiative to incorporate external paid digital educational resources, especially during the pandemic. In this study, we organized an exemplary questionnaire method to enable evidence-based decisions before providing paid e-learning resources to medical students.

**Methods:**

An online form was distributed to all registered medical students at a Turkish medical school, and the compiled responses were subjected to statistical analysis. The participants were queried about their general background, post-graduation plans, use of study materials, purpose and perceived benefits of utilizing paid e-learning resources, as well as any financial burden or opinions regarding the associated costs.

**Results:**

A total of 119 medical students participated in the online form. The findings revealed that 87% of the participants reported using paid e-learning resources for school exams, with 71.5% of them indicating an improvement in their exam scores. Approximately 26.1% of the participants did not specify any change. When asked to rate the cost of using paid e-learning resources on a scale of 1 to 10, the average score was 8.6 ± 1.58. Furthermore, 40% of the participants relied on repeated free demo sessions, while only 27% reported paying the fees associated with these resources.

**Conclusions:**

With the evolution of medical education, particularly in the aftermath of the pandemic, medical students are increasingly seeking to supplement their medical curriculum, pursue self-interests, and engage in extracurricular research by utilizing paid e-learning resources. However, the costs associated with these resources often prevent some students from fully benefiting from them. Therefore, it is essential for medical schools to make evidence-based decisions to support their students, recognizing that digitally available resources play an integral role in the assimilation of medical education.

## Introduction

Pre-clinical and clinical medical education is rapidly evolving due to factors such as the exponential growth of biomedical knowledge, the implementation of new pedagogical methods for adult learners, the impact of artificial intelligence, and the widespread adoption of digital technologies [1]. In addition to traditional resources like course lectures, practical skills lessons, tutorials, and textbooks, medical students are increasingly turning to external online educational materials, referred to as e-learning resources, which are available through paid subscriptions.

E-learning resources encompass various types, including question banks, video lectures, article-based resources, or a combination thereof. Students utilize these resources for different purposes, such as preparing for board exams or supplementing their studies in areas like anatomy, physiology, pathology, disease research, diagnostic skills, physical examination, and clinical competence [2,3]. Online paid e-learning resources offer medical students the flexibility and self-paced learning they seek.

E-learning resources empower users to take control of their learning, offering benefits such as a sense of autonomy and flexibility in the learning process [4,5]. Research has shown that self-directed learning through digital technology has been successfully integrated into medical curricula, leading to improved student performance and self-assessment of knowledge in countries like Germany [6,7]. The COVID-19 pandemic may be considered the triggering event in the shift to self-directed learning as educational institutions transitioned to virtual or hybrid models, emphasizing student-centered education [8]

The aim of this study is to evaluate the reasons behind medical students’ use of paid e-learning resources and determine their preferred types of resources. The findings can guide program directors and coordinators in better understanding medical students’ approach to e-learning resources.

## Materials and methods

### Population

The total duration of medical education in Turkey is six years instead of four years of pre-med and four years of medical education. The first three years usually cover basic sciences and preclinical knowledge, the fourth and fifth years generally consist of several clinical clerkships and the sixth year is the internship year. In the exemplary medical school, these periods are labeled as Phase 1, Phase 2, and Phase 3 in respective order. In this study, the enrolled medical students were presented with an online survey available both in Turkish and English prepared by the authors of this study. The survey was available to be completed by all willing 455 Phase 1, 2, and 3 students from 13 to 26 June 2022, and students were notified about the online survey through email announcements.

There were no exclusion criteria specified for the participants, therefore a census sampling technique was used in this study. The total number of enrolled students was 119, consisting of 54 female and 65 male students. There were three sections to the survey. The first section explained the study’s general purpose, confidentiality, and required participants to select an option confirming their agreement and consent for data analysis of their anonymized responses prior to the rest of the survey.

In the second part of the survey, participants were asked multiple-choice questions about their year in medical education, gender, their graduated high school background, their GPA, involvement in research, and plans for undergoing a residency program in a different country. In this study, medical students’ overall GPA was divided into 5 brackets based on the grading system of the school of medicine. These brackets covered overall GPA scores of 0–2.49, 2.50–2.99, 3.00–3.49, 3.50–3.74, and 3.75–4.00 in ascending order.

In the final section of the survey, participants were required to respond to a series of carefully designed questions, which were presented in the form of semi-closed, multiple-choice, or Likert-scale formats. These questions were aimed at gathering insights into the participants’ overall use of educational materials, including their usage of institutionally subscribed resources, paid e-learning resources, any financial burden associated with their use of e-learning resources, the reasons behind their use of specific resources, and the institutionally provided e-learning resources that they have utilized. These questions were asked to help MESC, a student-body commission, to better understand their classmates’ needs or study profiles so that if reasonable, a proposal to be made to the decision-makers to consider re-evaluating currently used institutionally subscribed resources, and determine whether the findings warrant any incorporation or substitutions with other e-learning resources to better tailor to their students. The potential financial burden of third party-resources was investigated and the students were also requested to evaluate this burden according to their benefit-to-cost ratio.

When considering the voluntary use of e-learning resources in this cross-sectional study, it considers that the resource should be capable of supplementing or assisting medical students mainly in their university’s medical education, or for extracurricular purposes: such as research, or when preparing for board examination(s).

Furthermore, the student or user of the e-learning resource is required to make a one-time or continuous financial investment in order to access its content. Therefore, free-to-access platforms or tools, such as YouTube channels, are not considered e-learning resources for no involved financial burden or students’ desire to unconventionally access the resource could be comparably analyzed in the scope of this study. Finally, the user should be able to interact with the e-learning resource in the form of an online platform; therefore, the user should be able to either search, request, command, or navigate the online platform to obtain their desired information or results. To further clarify, digital or hard-copy textbooks are not considered e-learning resources in this study; examples could include USMLE Step 1 high-yield revision textbooks or clinical medicine pocketbooks.

This study categorizes paid e-learning resources depending on their main intended use by medical students. Classifying educational e-learning resources is essential to better understand a student’s justification for using them, and how medical schools can take a more holistic approach to their students’ needs. Regarding this matter, this study has subclassified e-learning resources’ main intent of use into the following categories: 1.) Preparatory resources, intended solely for board exam preparation, examples commonly include question banks or short and high-yield content video-lectures tailored to preparing for the USMLE 2.) General extracurricular content, where the resources assist in assimilating medical education, and are not specifically tailored to pre-defined learning objectives of a specific curriculum or exam, examples commonly include an online medical education platform with video lectures.

Furthermore, paid e-learning resources will also be categorized based on their common mode of presentation of their educational content to their user. Regarding this matter, this study has subclassified e-learning resources’ method of presentation into the following categories:1.) Visual presentation, where an e-learning resource portrays their content mainly through video and image-based media 2.) Written presentation, where an e-learning resource portrays its educational content mainly through readable texts or material.

An application was submitted and approved by the institutional review board’s ethics committee. The students’ participation was purely voluntary, as explained in the first part of the survey. Informed consent was obtained electronically from all participants, documented through a digital consent form that participants were required to complete before proceeding with the survey. Since the study did not include minors, no parental or guardian consent was necessary. All data were kept confidential, in accordance with the ethics committee’s guidelines.

### Statistical analysis

The statistical analyses were performed using RStudio: Integrated Development for R (2021) with R: A language and environment for statistical computing (version 4.1.0). A p-value less than 0.05 was considered significant. The normality, kurtosis, and skewness of continuous data were assessed. The continuous variables are presented either with mean and standard deviations while categorical and ordinal variables are presented with frequencies and percentages. Percentages were calculated for all relevant variables to provide further insights into the data distribution.

Group comparisons for non-parametric continuous variables were conducted with the Kruskal-Wallis test and Mann-Whitney-U test for post-hoc analyses. The categorical variables were analyzed with the chi-square test and Fisher’s exact test for post-hoc analyses. A multinomial log-linear model was fitted for school grades. Correlations between e-learning resource preferences and academic characteristics were investigated.

## Results

From 25.5% of all medical students completed the survey and the respondents of this study were a relative representation of the medical school demographics as gender ratios and year distributions were not significantly different compared to the population (p > 0.05). Demographic characteristics investigated in this study were gender, year, GPA bracket, type of high school, and research participation status. When questioned about their preferred country for residency training, only 25.8% of respondents preferred Turkey while 74.2% preferred another country. Table 1 shows the distributions of respondents in several distinctive characteristics that were investigated in this study.

**Table 1:** The characteristics of the respondents (n = 119).

Variables	Number and percentage
**Sex**	
Male	65 (54.6%)
Female	54 (45.4%)
**Year**	
1	26 (21.8%)
2	23 (19.4%)
3	25 (21%)
4	20 (16.8%)
5	17 (14.3%)
6	8 (6.7%)
**Background**	
Local Students	93 (78.2%)
International Students	26 (21.8)
**Grade Point Average**	
3.75–4.00	42 (35.8%)
3.5–3.74	27 (23.1%)
3–3.49	33 (28.2%)
2.5–2.99	14 (12%)
2–2.49	1 (0.9%)
**Research Group Involvement**	
Involved in Research	81 (68%)
Not Involved	38 (32%)
**Usage of Premium Resources Provided for Free by University Classified by Method**	
Written	89 (74.8%)
Visual	74 (62.2%)
**Spending on Third-Party Resources (Annual, US Dollars)**	
NA	43 (36.1%)
< 5	21 (17.6%)
5 ≤ … <200	34 (28.6%)
≥200	21 (17.7%)
**Requested Premium Third-Party Resources** **Classified by Method**	
Written	82 (68.9%)
Visual	114 (95.8%)
**Classified by Goal**	
Board Exam Oriented	117 (98.3%)
Lecture Oriented	48 (40.3%)
**Residency Preferences**	
Turkey	30 (25.2%)
United States of America	40 (33.6%)
Germany	20 (16.8%)
United Kingdom	12 (10%)
Others (Canada, Switzerland)	8 (6.7%)
NA	9 (7.5%)
**Reasons to Use Premium Third-Party Resources**	
Board Exams	24 (20.1%)
Scientific Studies	33 (27.7%)
Complementary to Lectures	101 (84.9%)
Self Interest	38 (31.9%)
**If You Use Third-Party Resources Complementary To Lecturers, Do You Think It Is Beneficial? (N = 101)**	
Yes	63 (62.4%)
Maybe	23 (22.8%)
No	2 (2%)
NA	13 (12.8%)
**How expensive do you find membership services?**	
**(1**–**10)**	
1	1 (0.8%)
2	0 (0%)
3	0 (0%)
4	1 (0.8%)
5	3 (2.5%)
6	3 (2.5%)
7	13 (10.9%)
8	25 (21%)
9	18 (15.2%)
10	43 (36.2%)
NA	12 (10.1%)
**How do you access other paid third-party resources?**	
Scholarship	11 (9.2%)
Paying the price	31 (26.1%)
Occasionally Breaching Access Protocols	19 (16%)
Using demo accounts many times	47 (39.5%)
Account Sharing	9 (8%)

Even though there were no significant differences for countries of choice for residency training in different years or backgrounds when grouped into students’ phases, significantly more medical students from Phase 1 preferred the USA for residency training. (p = 0.033)

While all survey respondents reported using paid e-learning resources, 87% of medical students reported using e-learning resources for school exams, 32.7% for personal interest, 28.4% for scientific research, and 20.6% for board exams. From 71.5% of students who reported using e-learning resources for school exams also reported an increase in their exam scores, while 26.1% did not specify any changes, and 2.2% reported a decrease.

When asked about the perceived expensiveness of paid e-learning resources on a 1–10 Likert scale, 74.1% of students reported scores of eight, nine, and ten with an average score of 8.6 ± 1.58 in a scale of 1 to 10 with 1 being cheap and 10 being expensive. 40% of medical students reported relying on repeated free demo sessions, 27% reported paying the fees, 16% reported occasionally breaching the access protocols, 9% reported having access covered by scholarships and 8% reported using a single account with their friends when accessing paid e-learning resources.

There were no significant differences in medical students’ preferences for visual, written or board exam-oriented e-learning resources, independent of being institutionally subscribed, depending on gender, year, GPA bracket, background, or research participation status (p > 0.05).

## Discussion

Traditional medical education was greatly hampered during the Covid-19 pandemic and blended learning and e-learning presented themselves as alternative teaching-learning methods. E-learning is referred to as using online tools to improve performance and increase knowledge, while blended learning combines face-to-face learning and e-learning modalities. With the use of e-learning technologies, learners have been able to customize their experiences to match their own learning goals by having control over the content, learning process, speed of learning, and use of their time [9].

In 2020, Vallée et al. stated that when compared to conventional learning in health education, blended learning regularly outperformed traditional learning in terms of knowledge outputs [10]. The findings presented in their research align with our findings, where the majority reported using e-learning resources for school exams and scoring better in these exams. Innovations in e-learning technologies along with the quick adoption of virtual learning as a result of the Covid-19 pandemic hint at a revolution in education by allowing for personalized learning. Additionally, online e-learning resources can enable tracking of missed or poorly understood subjects and can provide individualized study strategies, enabling better time management by focusing on specific learning outcomes [1,11]. More research describing institutional experiences with different platforms is necessary to investigate the effectiveness of various blended learning design variants.

The incorporation of e-learning into medical education has already initiated a change toward using adult learning theory, in which educators take more active roles as facilitators of learning and evaluators of competency rather than just information providers [1,12]. Additionally, medical students are expected to be self-directed lifelong learners as medical education shifts toward competency-based models. It is critical to understand what technology and platform medical students prefer utilizing for self-directed learning and what variables contribute to students’ self-initiated technology use and possibly their consequential improved performance [13]

With the increasing availability to diverse types of paid e-learning resources, some university libraries subscribe to these multimedia resources to provide access to the faculty responsible for education and training in efforts to support their students’ learning process. Offering paid e-learning resources to university students free of charge via institutional subscriptions by libraries could result in their extensive use by today’s medical students, as they are technologically skilled and possibly will leverage available mobile applications of innovative course resources to enhance their assigned curriculum’s learning experiences. Additionally, the emerging accessibility of these e-learning resources through mobile applications has made it easier for students to access their educational content presented either in the form of animations, videos, 3D images, articles, or pictures. Considering medical students’ preference to use high-yield and easily accessible content, mobile applications increase the e-learning resources potential alongside traditional education resources [3].

Although there is some research exploring e-learning resources’ effects on medical education and student performances, not many papers compare different resources, let alone classify them based on the provided type of informative content or how it is presented. Bauzon et al. assessed the perceived preparedness of students that used different resources. Their study investigated online video-based supplemental curricula, question banks, visual learning platforms, and flashcard review programs as well as books. As free-to-use platforms and physical materials were not included in this study, we opted for a similar classification. In this study, we classified resources based on the type or presentation of material they provided. Some e-learning resources rely more on visual content, whereas some rely more heavily on written materials, and some are intended as board exam preparation/materials while others are more complementary to medical school lectures’ learning objectives. It is important to note that there are e-learning resources that provide both kinds of content but with one aspect more prominent than the other. Hence, even though there are many accepted types of resources, there is no one-fitting-all approach to classify them. In this regard, educational institutions could benefit by analyzing their students’ learning profiles with all its dimensions. This analysis can guide the institutions to assess when intending to invest in resources that could better suit their educational needs.

It is observed that aspiring medical students from certain parts of the world seek to complete their residency training in foreign countries due to a variety of reasons. This trend has been outlined by several authors in the past years: some flee from domestic instability; some seek better living standards, but some simply desire to receive better training or to obtain research opportunities they may not have had in their home country. It should be noted that providing resources such as USMLE question banks could be a double-edged sword. While it enhances students’ exam preparation and readiness for opportunities abroad, it also potentially exacerbates the movement of graduates away from their home countries, contributing to physician shortages in these regions [14–17].

This could reflect the concept that digital e-learning resources are not just benefiting their users to assimilate their knowledge of medicine but can also serve as a crucial tool bridging medical students intending to grow beyond their regional horizons which were initially limited or bounded by their dependence on locally available resources. Medical students intending to emigrate and practice abroad as International Medical Graduates (IMGs) face unique challenges in adapting to the constantly fluctuating approach or understanding of medicine, such as modernized approaches to diseases or updated meticulous guidelines that may not have been covered in their home country’s medical education. For example, when examining the ratio of IMGs to US-Graduated physicians practicing medicine in the USA, the ratio has remained more or less the same; however, this also reflects that the total number of IMGs has been increasing in conjunction with the number of medical personnel [18]. In our study, one-third of our respondents expressed a desire to complete their residency training in the USA, which would require taking the USMLE while preparatory online materials for board exams have been found to be an important component of medical student debt [18]. A study conducted by the American Medical Students Association in collaboration with Wolters-Kluwer found that both American and international medical students were spending more and more on online e-learning resources [19]. The average spending of international students sometimes exceeded that of their American peers, despite possibly not having the same purchasing power. A majority of this expenditure was seen spent on board review/prep materials totaling $7,500 on average before the COVID-19 pandemic [20]. As the pandemic forced all educational institutions to change their didactic methods, the integration of other online learning materials into medical school curricula has accelerated ever since [21].

While it is undeniable that online learning materials are becoming an increasingly prevalent part of one’s medical education, because the aforementioned costs of such platforms are one of the most important factors for students choosing paid e-learning resources, we believe that medical schools ought to first investigate students’ preferences before providing institutional subscriptions to better suit students’ needs in order to alleviate some of the financial burden associated with their usage [22]. We have found that the vast majority (75%) of medical students perceived e-learning resources to be rather expensive. When asked about how they financed or gained alternative means to access their e-learning resources, only 27% of the participants in this study reported individually paying for the required fees. Repeated free demo sessions and occasionally breaching access protocols accounted for 40% and 16% respectively, indicating less-than-ideal circumstances when accessing and benefitting from e-learning resources. Medical schools providing institutional subscriptions to high-demand resources can greatly improve equity in medical education by removing financial obstacles to what is becoming an increasingly important aspect of medical education [22]

Furthermore, there is an emerging concern that the extensive reliance on high-yield exam questions, such as those offered by many paid e-learning resources, might limit students’ understanding of broader medical knowledge that is essential for high-quality patient care. Medical schools and educators should be cautious not to encourage students to focus exclusively on exam preparation at the expense of developing well-rounded clinical skills and knowledge necessary for patient care. Therefore, a balance between providing resources for exam success and promoting comprehensive learning is crucial [23–25].

## Summary

Based on the results of this survey, a proposal or request will be made to the decision-makers to re-evaluate currently used intuitionally subscribed paid e-learning resources in relation to the findings of this survey to see if any amendments are warranted. Students’ preferences may differ from one institution to the other, hence, the main lesson to be taken from this research is that a better understanding of students’ needs or study preferences may benefit medical schools greatly, especially in this new age of medical education and ever-evolving digital resources. The questionnaire used in this study has been answered by the students of a single medical school and may not represent all medical students in Turkey.

Nevertheless, this study further cements the notion of medical schools’ responsibility in connecting with their students; therefore, this research can guide researchers in different institutions in gaining a better understanding of their medical students’ perspectives toward paid e-learning resources in order to make evidence-based decisions.

## Conclusion

Paid e-learning resources have already become an integral part of medical education, and medical schools ought to ideally make evidence-based decisions that enable equitable, institutional subscription to paid e-learning resources for their students while ensuring a balanced approach that supports broad-based medical knowledge in addition to exam success.
